# Towards Tuning the Mechanical Properties of Three-Dimensional Collagen Scaffolds Using a Coupled Fiber-Matrix Model

**DOI:** 10.3390/ma8085254

**Published:** 2015-08-20

**Authors:** Shengmao Lin, Lauren A. Hapach, Cynthia Reinhart-King, Linxia Gu

**Affiliations:** 1 Department of Mechanical and Materials Engineering, University of Nebraska-Lincoln, Lincoln, NE 68588-0656, USA; E-Mail: linshengmao@gmail.com; 2 Biomedical Engineering, Cornell University, Ithaca, NY 14853, USA; E-Mails: lah299@cornell.edu (L.A.H.); cak57@cornell.edu (C.R.-K.); 3 Nebraska Center for Materials and Nanoscience, Lincoln, NE 68588-0656, USA

**Keywords:** collagen scaffold, fiber-matrix interaction, glycation, collagen concentration, computational biomechanics

## Abstract

Scaffold mechanical properties are essential in regulating the microenvironment of three-dimensional cell culture. A coupled fiber-matrix numerical model was developed in this work for predicting the mechanical response of collagen scaffolds subjected to various levels of non-enzymatic glycation and collagen concentrations. The scaffold was simulated by a Voronoi network embedded in a matrix. The computational model was validated using published experimental data. Results indicate that both non-enzymatic glycation-induced matrix stiffening and fiber network density, as regulated by collagen concentration, influence scaffold behavior. The heterogeneous stress patterns of the scaffold were induced by the interfacial mechanics between the collagen fiber network and the matrix. The knowledge obtained in this work could help to fine-tune the mechanical properties of collagen scaffolds for improved tissue regeneration applications.

## 1. Introduction

Three-dimensional (3D) scaffolds are commonly used as microenvironments for regulating cellular functions and supporting tissue regeneration *in vitro* as well as *in vivo* [1,2]. Their mechanical characteristics have been acknowledged as important factors in cell functions including growth, migration, proliferation, and apoptosis [3]. Three dimensional cell culture systems have been gaining more attention due to their capacity to better capture complex cell-scaffold interactions compared to two-dimensional platforms [4]. Numerous hydrogel systems have been utilized for 3D cell culture to better understand the role of scaffold mechanics in mediating cell behavior within certain environments [5,6,7]. Specifically, collagen hydrogels are a viable scaffold for regenerating tissues such as skin [8], cartilage [9], tendons [10], and blood vessels [11]. The microstructure and stiffness of collagen gels can be tuned using various techniques including altering the collagen concentration [12], changing the extent of crosslinking using techniques such as glycation, commonly utilizing glucose or ribose as reducing sugars [13], and adding synthetic polymers such as polyethylene glycol (PEG) [14] or natural proteins such as agarose [15]. However, it remains difficult to tune individual scaffold properties without altering the microstructure of the scaffold.

Mason *et al.* recently demonstrated that non-enzymatic glycation can be used to control collagen scaffold stiffness without significant microstructural changes within the range of 0–100 mM ribose [5]. Non-enzymatic glycation is the result of covalent bonding of a protein with a sugar molecule, such as glucose or ribose. In this case, during non-enzymatic glycation, the ribose interacts with amino groups on collagen to form Schiff bases that can rearrange into Amadori products [16]. These Amadori products subsequently form advanced glycation end products (AGE) that accumulate on collagen. Results showed that the compressive modulus of collagen scaffolds were increased threefold after glycation, along with a significant increase in cell growth and spreading. Even though Mason *et al.* [5] has only characterized gels with the collagen concentration of 1.5 mg/mL, it is interesting to observe that the gel modulus increased without significant microstructural changes. This led to our hypothesis that the change in gel modulus is due to altered interfacial mechanics between individual collagen fibers and their surrounding matrix. Since the collagen fiber network does not show significant changes, different levels of ribose used for non-enzymatic glycation results in changes in the shear modulus of the matrix.

The goal of this work is to develop a computational framework for capturing the above mentioned experimental results [5] and provide additional insight on the fiber-matrix interface beyond the discrete experimental datasets. Here, we model the detailed fiber-matrix interactions following non-enzymatic glycation of the collagen scaffold. In addition, three different collagen concentrations were investigated to separate the coupled effect of collagen concentration and glycation on the mechanics of the resulting collagen scaffold. The knowledge obtained in this work could help to fine-tune the mechanical properties of collagen scaffolds for controlling cellular functions and ultimately lead to better tissue regeneration.

## 2. Materials and Methods

**Figure 1:**
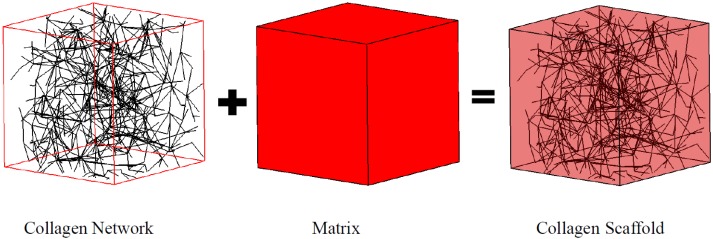
Collagen scaffold represented by the coupled fiber-matrix model.

In this work, a collagen fiber network was modeled as a Voronoi diagram (Figure 1), which has demonstrated its utility elsewhere [17,18]. Briefly, Delaunay triangulation [19] was created by linking randomly seeded nodes within a representative volume element (RVE) using Matlab (Natick, MA, USA). A total of 698 fibers with a diameter of 62 nm were generated within the 10 μm square cubic RVE to mimic the fiber microstructure for a collagen concentration of 1.5 mg/mL. The fiber dimensions were adopted based on the measurement from collagen gel polymerized at 37 °C and pH 7.4 [20]. The same fiber diameter was also used in our previous study [21], which configured a significantly different fiber network compared to the current work. The collagen fiber network herein was designed to mimic the fiber entanglement rather than the exogenous cross-linker as in our previous work. Each fiber was meshed with 100nm beam elements and the Young’s modulus was adopted as 50 MPa [22]. The fiber network was embedded in the matrix to formulate the RVE as illustrated in Figure 1.

The matrix was meshed with 125,000 eight-node brick elements and considered as an incompressible neo-Hookean solid [23] as defined by its strain energy density function *W*:

(1) $$W=\frac{\text{μ}}{2}(I_{1}-3)$$

where μ is the shear modulus and *I*_1_ is the first invariant of the right Cauchy-Green deformation tensor. Per our hypothesis, shear moduli of the matrix, *i.e.*, 11 Pa, 30 Pa, and 50 Pa, were reverse fitted corresponding to three levels of ribose concentration (0 mM, 50 mM, and 100 mM) as used in the experimental work by Mason *et al.* [5]. A 5% compressive strain used in the experimental protocol was also applied on one surface of RVE for observing the mechanical behaviors of collagen scaffold. The RVE models were solved using ABAQUS 6.12 (Simulia, Providence, RI, USA).

## 3. Results

**Figure 2:**
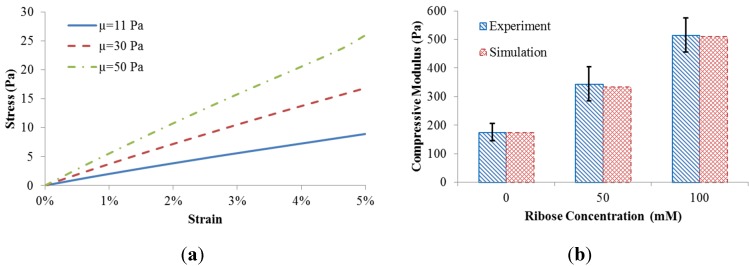
(**a**) Scaffold behavior in response to matrix shear modulus; (**b**) Model validation.

The coupled fiber network and matrix model resulted in different scaffold behavior when the matrix shear modulus was adjusted (Figure 2a). The compressive stress of the scaffold was calculated by the predicted reaction force divided by the side area. The equilibrium compressive modulus of the scaffold was obtained by a linear fit to the stress-strain datasets.

To match the measured equilibrium compressive modulus [5], the matrix shear moduli (µ = 11 Pa, 30 Pa, and 50 Pa) were correlated with levels of ribose concentration (0 mM, 50 mM, and 100 mM) used for non-enzymatic glycation. The comparison between our computational predictions and the published experiments [5] is shown in Figure 2b. The good match indicates that different levels of ribose indeed result in altered shear modulus of the matrix. As such, experimentally observed gel stiffening with altered ribose concentration can be explained computationally based on changes to matrix rather than collagen fiber microstructure.

**Figure 3:**
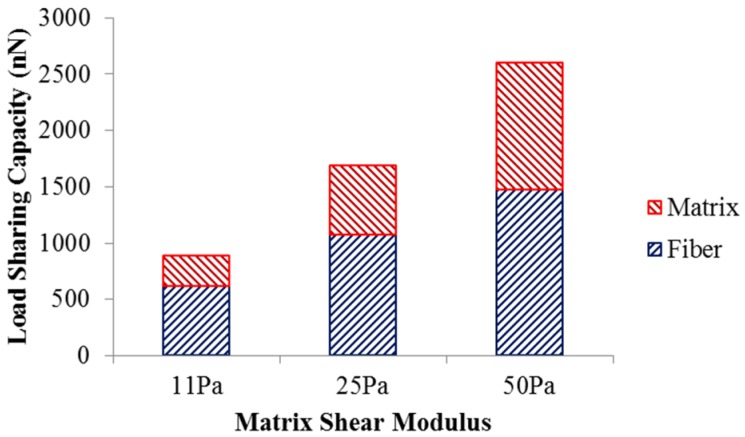
Load sharing capacity of scaffold.

The load sharing in the scaffold with different matrix shear moduli is illustrated in Figure 3. As the matrix shear modulus is altered from 11 Pa to 50 Pa with the fiber network structure intact, more load is required to deform the scaffold to achieve 5% strain. The load undertaken by the matrix increased from 270.9 nN to 1124 nN, while the load shared by the fiber network increased from 619.8 nN to 1474 nN. This also led to an increase in the matrix’s load-sharing from 30% to 43%, and a reduced percentage of load-share for the fiber network. The increased load-sharing capacity of the matrix could be explained by the increased matrix modulus ratio, *i.e.*, matrix modulus over the fiber modulus.

**Figure 4:**
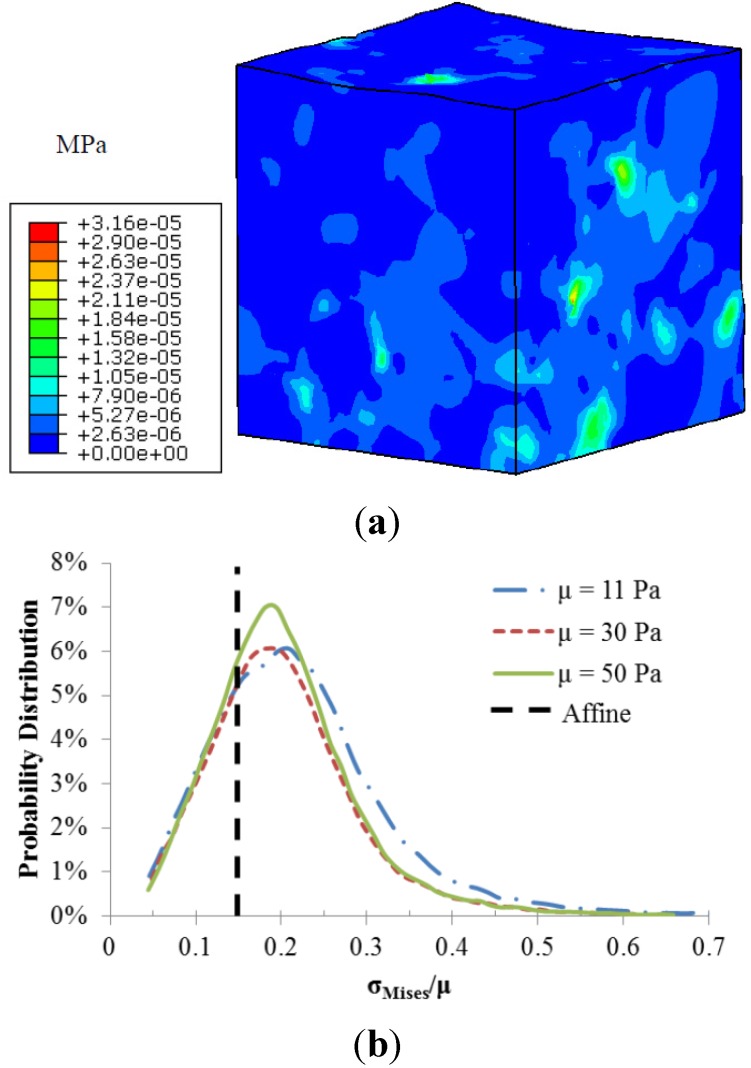
(**a**) Von-Mises stress distribution of the matrix with shear modulus of 11 Pa; (**b**) Probability distributions of normalized matrix stress.

The fiber-matrix interaction also resulted in heterogeneous stress distributions as illustrated in Figure 4. Stress concentrations occurred at the regions around the fiber intersections. The calculated local matrix stress gradient increases with a stiffer matrix. However the sensitivity of this local stress gradient to the matrix modulus diminishes with a stiffer matrix. The probability distributions of normalized matrix stress (σ_Mises/_μ) depicted in Figure 4b were also used to demonstrate the role of matrix stiffness in the matrix stress inhomogeneity. The black dotted line is the uniform stress distribution in a pure matrix without fibers, also referred to as affine deformation. It is clear that the increased matrix shear modulus led to a shift in the probability distribution closer to the affine case, indicating reduced stress inhomogeneity.

**Figure 5:**
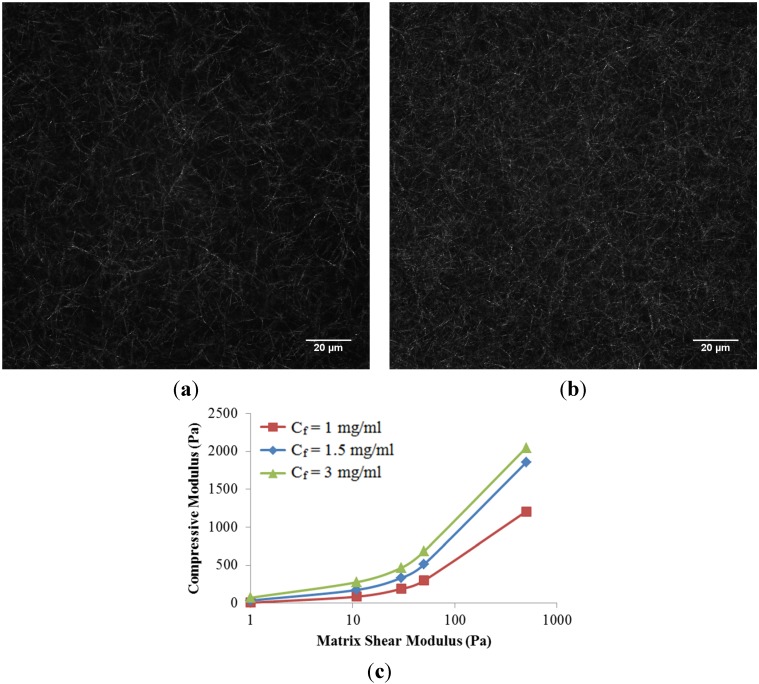
Confocal reflectance microscopy images of scaffold at the collagen concentration (*C*_f_) of (**a**) 1.5 mg/mL, and (**b**) 3.0 mg/mL, respectively; (**c**) Compressive Modulus of collagen scaffolds in response to collagen concentrations and matrix shear modulus.

The role of collagen concentration on the mechanical response of the collagen scaffold is also investigated in this work. Two additional collagen concentrations (1 mg/mL and 3 mg/mL) were compared to the baseline case at 1.5 mg/mL. Higher concentrations of collagen resulted in a denser fiber network (Figure 5a,b), as expected. In addition, two additional matrix shear moduli (0 and 500 Pa) were considered to represent an isolated fiber network without matrix and a much stiffer matrix case, respectively. Here, we have assumed that fiber microstructure is kept intact at a higher matrix shear modulus, *i.e.*, the higher ribose concentration used for non-enzymatic glycation. It is observed that the compressive modulus of the scaffold increased nonlinearly with collagen concentrations as well as matrix shear modulus (Figure 5c). The nonlinearity created by increasing the matrix shear modulus alone could be explained by the shift from fiber dominated mechanics to matrix dominated mechanics. The increased scaffold stiffness due to increased collagen concentration alone is due to the compact fiber network. If the matrix shear modulus below is limited to 50 Pa per the published experiments [5], a linear relationship with a correction coefficient (*R*^2^) of 0.9645 among the scaffold compressive modulus (*E*, in Pa), matrix shear modulus (µ, in Pa), and collagen concentration (*C*_f_, in mg/mL) was then obtained and summarized as

*E* = 8.942µ + 105.3*C*_f_ − 134.2 (2)

This empirical equation could be used to fine-tune the configuration of scaffold.

## 4. Discussion

A coupled fiber-matrix finite element model was developed in this work and used for predicting the mechanical response of collagen scaffolds with different levels of non-enzymatic glycation and collagen concentrations. The collagen network was modeled as a Voronoi network embedded in a matrix. The macro compressive modulus of the scaffold increased three-fold by tuning the ribose concentration from 0 mM to 100 mM, corresponding to the matrix shear modulus from 11 Pa to 50 Pa. This correlation is based on the experimental observations [5] that the above mentioned range of ribose used for non-enzymatic glycation did not alter the scaffold microstructure, *i.e.*, the fiber network remains the same. This indicated that the three-fold scaffold stiffness increase could be fully due to the matrix stiffness, rather than the fiber network. The predicted results along with our computational framework were validated by published experiments (Figure 2). The validated model could then be used to inspect the mechanism of the altered fiber-matrix interactions as well as to identify the factors regulating the scaffold mechanics.

The altered scaffold stiffness could be explained by the load sharing capacity of the matrix and fiber network as well as the stress heterogeneity induced by the fiber-matrix interactions. Even though a larger force is required to deform the scaffold to its 5% strain with increased matrix stiffness, a large percentage of the extra load is shifted to the matrix (Figure 3). This also led to the altered interfacial mechanics between collagen fibers and matrix, which resulted in heterogeneous scaffold mechanics (Figure 4). Stress concentrations were found at the fiber-matrix interface, especially at fiber clusters where several fibers were entangled. However, this matrix stress heterogeneity reduced with a larger matrix modulus. These local scaffold mechanics will likely affect cell behaviors, such as cell migration towards regions of stiffness [24]. The scaffold heterogeneity was also recognized in previous numerical studies [18,25] and needs to be further investigated.

The roles of collagen concentration, *i.e*, the fiber network density ribose concentration, and the matrix modulus, were isolated to better understand the contribution from each element to the mechanical behavior of scaffold (Figure 5 and Equation (2)). The increase in either matrix modulus alone or collagen concentration alone has demonstrated its capacity to stiffen the scaffold as well as to diminish the local stress heterogeneity. Both individual elements led to reduced material mismatch between the fiber network and matrix. In addition, the scaffold was more sensitive to the alteration of collagen concentrations. This could be explained by the dominant role of the fiber network in sharing the scaffold load. It should be noted that Equation (2) is valid for a ribose concentration below 100 mM, *i.e.*, the matrix shear modulus below 50 Pa. This is due to a larger ribose concentration, which also induced the microstructural changes in the fiber network, such as increased crosslinking, and reduced entanglement between fibers. These alterations together led to a nonlinear behavior of scaffold modulus related to a large range of ribose concentrations [5]. The predictive model needs to consider the competitive effects of both the altered fiber network and matrix stiffening on the scaffold modulus. Specifically, the fiber thickening resulted in an increased scaffold modulus, while conversely, the reduced fiber entanglement led to a smaller scaffold modulus. If the fiber network was assumed unaltered by a wide range of ribose concentrations, the nonlinear behavior in Figure 5 was then observed.

## 5. Conclusions

In this study, a coupled fiber-matrix numerical model was developed to predict the mechanical response of collagen scaffolds subjected to various levels of non-enzymatic glycation and collagen concentrations. The scaffold was simulated using a Voronoi network embedded in a matrix. The constructed fiber network density was regulated by the collagen concentration, while non-enzymatic glycation led to increased matrix stiffness. The computational model was validated with previously published experimental data. Results show that scaffold modulus was linearly correlated with both matrix stiffness and collagen concentration for a ribose concentration below 100 mM. This correlation became highly nonlinear, where a larger ribose concentration induced microstructural changes in the fiber network. More crosslinking between fibers were also speculated to contribute to the glycation induced scaffold stiffening. This aspect was not explicitly included in this work due to lack of experimental data. Appropriate experiments need to be designed to quantify the role of glycation on both matrix stiffness and crosslink density.

In summary, the developed models offer an effective means to integrate experimental datasets and facilitate investigation of the scaffold mechanics where experimentation is inefficient. The detailed fiber-matrix interaction could be used to guide the design of collagen scaffolds. More modeling details such as fiber curvature and its nonlinear material properties could also be included for better inspection of the interfacial mechanics. The insight gained in this work could lead to a better understanding of how to fine tune the mechanical properties of collagen scaffolds for optimal tissue regeneration applications. The model could also be extended to study the cell-scaffold interactions with independent control of fiber microstructure and local stiffness.
